# Effect of Periodontal Treatment in Patients with Periodontitis and Diabetes: Review of Systematic Reviews with Meta-Analyses in the Last Five Years

**DOI:** 10.3390/healthcare12181844

**Published:** 2024-09-14

**Authors:** Nansi López-Valverde, José Antonio Blanco Rueda

**Affiliations:** 1 Department of Surgery, University of Salamanca, 37008 Salamanca, Spain; jablancor@usal.es; 2 Biomedical Research Institute of Salamanca (IBSAL), 37007 Salamanca, Spain

**Keywords:** periodontitis, diabetes, periodontal treatment, humans, general overview

## Abstract

Background: Periodontitis is a chronic infectious–inflammatory pathology, with a high prevalence, which destroys the dental support and, if left untreated, leads to tooth loss. It is associated with other pathologies, particularly diabetes mellitus. Objectives: Our objective was to conduct a review of systematic reviews with meta-analyses to determine the evidence for periodontal treatment on periodontitis and diabetes. Second, we assessed the risk of bias and methodological quality using the AMSTAR-2 and ROBIS tools. Methods: We performed bibliographic searches in PubMed/Medline, Embase, Cochrane Central, Dentistry & Oral Sciences Source databases and in the Web of Science (WOS) scientific information service to identify systematic reviews with meta-analyses from the last five years. Results: Eighteen studies that met the inclusion criteria and evaluated 16,247 subjects were included. The most studied parameters were probing pocket depth, clinical attachment level, bleeding on probing and the glycated hemoglobin. Most of the included meta-analyses evaluated adult patients with periodontitis and type 2 diabetes mellitus (T2DM). Most of the meta-analyses considered and assessed by AMSTAR-2 showed significant methodological errors. The risk of bias was the domain with the worst assessment with the ROBIS tool. Conclusions: Despite the weaknesses of the included meta-analyses in terms of methodological quality and the risk of bias, periodontal treatment and DM treatment appear to contribute to improved clinical outcomes in a bidirectional manner between periodontitis and DM.

## 1. Introduction

Periodontitis is a chronic inflammatory disease of infectious origin and associated with the accumulation of dental microbial biofilm, generally caused by the progression of untreated gingival inflammation, which affects the supporting structures of the tooth and is manifested by the progressive destruction of the periodontal ligament and alveolar bone due to proinflammatory cytokines inducing bone resorption, such as Interleukin 1 beta (IL-1β) and tumor necrosis factor (TNF-α) [1].

The development of the disease is considered to be conditioned by complex interactions between the specific pathogens, the host response, the individual’s genetic condition, and a number of risk and epigenetic factors [2].

It affects approximately 11% of the world’s population, which means more than 750 million individuals whose masticatory ability is impaired, with a negative effect on their quality of life [3].

In recent decades, numerous investigations have studied the association of periodontitis with other systemic pathologies, such as diabetes, cardiovascular diseases, metabolic bone pathologies, premature birth, and recently, Alzheimer’s disease, as well as with other inflammatory and oncological pathologies [4,5,6,7].

Diabetes is the pathology most closely related to periodontitis and it is well known that patients with this pathology, especially in uncontrolled situations, have a high risk of developing periodontitis; on the other hand, periodontitis negatively influences the glycemic control of diabetes mellitus. Diabetes is the pathology most closely related to periodontitis and it is well known that patients with this pathology, especially in uncontrolled situations, have a high risk of developing periodontitis; the risk of periodontitis is increased by approximately threefold in diabetic individuals compared with non-diabetic individuals. On the other hand, periodontitis negatively influences the glycemic control of diabetes mellitus. Most studies focus on type 2 diabetes mellitus (T2DM) as a risk factor for periodontitis; however, it is known that type 1 diabetes mellitus is also a risk factor [8].

Many studies have supported the benefits of periodontal therapy in reducing glycemic levels [9,10,11]; however, there is no unanimity on this matter: a recent Cochrane review showed only moderate certainty that the treatment of periodontitis improves glycemic control [12].

**Figure 1:**
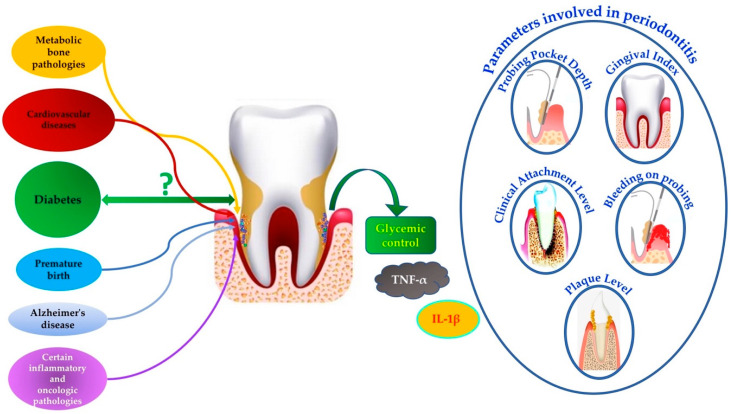
Representative graph of the relationship of periodontal disease with other pathologies.

The graph in Figure 1 represents the relationship of different pathologies with periodontal disease and the periodontal parameters involved.

Evidence-based care practice constantly requires a synthesis of the available scientific information, as well as of the knowledge gaps where research is needed. Systematic reviews (SRs) and meta-analyses (MAs) have increased in recent years and are considered the most reliable sources for therapeutic decision making [13]. They should be rigorous and transparent research and provide concrete answers to established research questions; however, they are not free of biases and errors [14,15,16]. 

In order for researchers to distinguish quality reviews, comprehensive critical appraisal tools have been developed. AMSTAR-2 (A MeaSurement Tool to Assess systematic Reviews) is an instrument that allows a detailed evaluation of SRs that include randomized or non-randomized studies of health interventions, in addition to providing guidelines on how to plan and conduct an SR, and is considered the most widely used quality assessment tool for SRs [17]. 

To assess the risk of bias in SRs, the ROBIS (Risk of Bias in Systematic Reviews) tool has been developed [18], which is summarized in three phases: (1) assess the relevance of the SR (optional), (2) identify concerns with the review process, and (3) judge the risk of bias in the review.

SRs and meta-analyses present a number of particularities in terms of their design and methodological tools to assess the risk of bias between intervention and control studies [19]. On the other hand, there are no specific tools that evaluate both the methodological quality and the risk factors of SRs and meta-analyses.

Therefore, the aim of our work was to determine the evidence of periodontal treatment on periodontitis and diabetes. Our secondary objective was to assess the methodological quality and risk of bias of the SRs using the AMSTAR-2 and ROBIS tools.

## 2. Materials and Methods

### 2.1. Registration and Description of Tools

This SR review was carried out following the established methodological proposals and was reported following the PRISMA 2020 reporting guidelines [20] (Supplementary Materials Table S1). The previously elaborated protocol was registered in INPLASY, registration number: INPLASY202450078; DOI number: 10.37766/inplasy2024.5.0078. 

The AMSTAR-2 tool [17] is presented in a questionnaire containing 16 domains with simple answers: “yes” when the result is positive, “no” when the information is insufficient and “partial yes” in cases where only partial compliance with the standard has been found. There are seven critical domains (domains 2, 4, 7, 9, 11, 13 and 15) with four confidence levels, high, moderate, low and critically low, and the identification of weaknesses in these domains can definitely decide the validity of an SR and its conclusions. The critical domains are as follows: 2, the registration of the protocol prior to the start of the SR; 4, adequacy of the literature search; 7, justification for exclusion of the primary studies; 9, the risk of bias of the primary studies; 11, adequacy of the meta-analysis methods; 13, the consideration of the risk of bias when interpreting the results; and 15, an evaluation of the presence and possible impact of publication bias. The non-critical domains are as follows: 1, the question includes the PICO components; 3, the reasons for the study design selected for inclusion in the SR are explained; 5, duplicate screening is performed; 6, duplicate data extraction; 8, an explanation of the included studies in sufficient detail; 10, reporting the financing of each primary study; 12, an assessment of the risk of bias on the results of the meta-analysis; 14, a correct explanation of the possible heterogeneity between primary studies; and 16, reporting conflicts of interest when conducting the SR. This tool does not generate an overall score. 

ROBIS [18] is the first tool developed and designed in a rigorous and specific way to assess the risk of bias in SR. Phase 1 assesses the relevance of the “target question”. Phase 2 identifies problems of bias in the review process in four areas, (i) study eligibility criteria, (ii) study identification and selection, (iii) data collection and study evaluation, and (iv) synthesis and results, while phase 3 considers whether the SR as a whole is at risk of bias. The overall assessment of the risk of bias of an SR in phase 3 uses the same structure as the separate domains of phase 2.

The SRs were assessed independently by two reviewers and a third external reviewer was used when necessary. The reviewers compared responses to each question and signaling domain. Disagreements were resolved by consensus.

### 2.2. Quality of Evidence

To assess the quality of the evidence, the GRADE criterion [21] was used, which establishes 4 categories: high, moderate, low, and very low. The high rating demonstrates high confidence in the coincidence between the real and estimated effect; moderate, moderate confidence; low, limited confidence; and very low, low confidence in the estimated effect.

### 2.3. Data Sources and Bibliographic Search

We conducted an electronic search in PubMed/Medline, Embase, Cochrane Central, Dentistry & Oral Sciences Source databases and in the Web of Science (WOS) scientific information service to identify SRs with meta-analyses published in English during the last five years, using the EndNote bibliographic reference manager (Clarivate Analytics).

The search strategy was designed with the help of an expert documentalist using the terms described in Table 1. We also searched the gray literature to obtain as much information as possible and to avoid bibliographic bias (GreyNet International). 

### 2.4. Inclusion and Exclusion Criteria 

Inclusion criteria were established according to the following guidelines: an SR with meta-analyses that included randomized clinical trials (RCTs) conducted in adult subjects (≥18 years) with a diagnosis of periodontitis and type 2 diabetes, undergoing surgical or nonsurgical periodontal treatment and/or adjuvant treatments and compared with untreated or placebo-treated subjects to observe effects on periodontal indices and/or the glycemic level.

We considered as exclusion criteria SRs that did not include meta-analyses; SRs that used only one database for record searches; and studies of predictive models or prognostic scales, since including this type of study would mean an unattainable bibliography to analyze in our study (Table 2).

### 2.5. Data Extraction

Two independent reviewers (NL-V and JAB-R) collected the titles and abstracts of the selected articles and entered them into an Excel spreadsheet and subsequently assessed the methodological quality of the included meta-analyses using the AMSTAR-2 tool and the risk of bias using the ROBIS tool. Discrepancies were resolved by consensus or by the intervention of an external evaluator. A narrative description of the extracted data was prepared and analyzed and an overlap-of-evidence document was prepared by cross-checking. A minimum overlap was considered to be between 0 and 5%, moderate between 6 and 10%, high 11–15% and very high when it exceeded 15%.

## 3. Results

**Figure 2:**
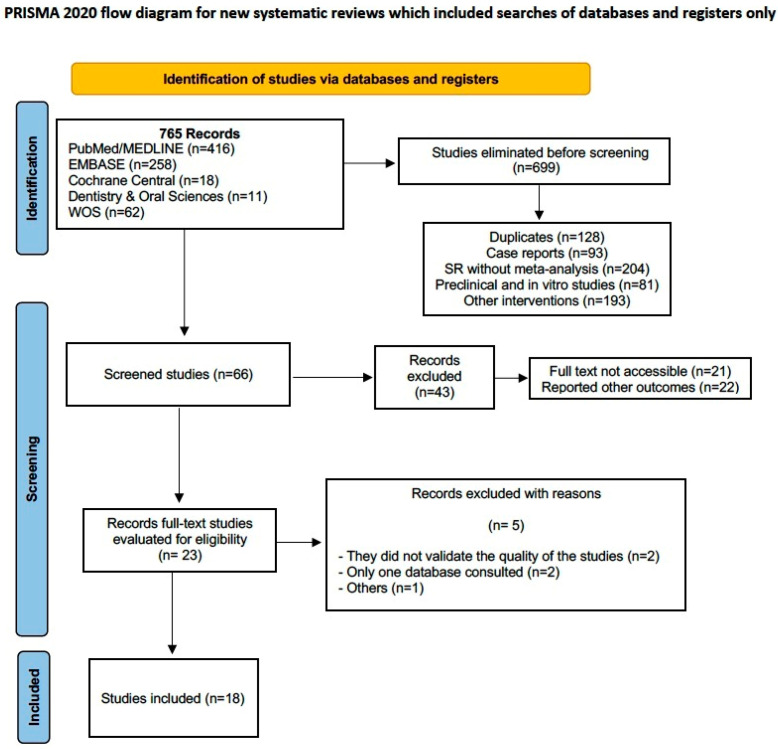
PRISMA 2020 flowchart describing the selection process of the included systematic reviews.

The main search identified 765 records in the last 5 years up to March 2024. The results were imported into Mendeley to remove duplicate and non-useful records for our study, resulting in 66 records for analysis. Subsequently, 43 were removed due to full-text inaccessibility and different results being reported, leaving 23 full-text records for assessment. Finally, for different reasons, 5 more were removed, resulting in 18 studies for inclusion [22,23,24,25,26,27,28,29,30,31,32,33,34,35,36,37,38,39] (Figure 2, Flow diagram).

### 3.1. Characteristics of Included Meta-Analyses

The included meta-analyses evaluated, in turn, 316 RCTs incorporating a total of 16,247 individuals. The meta-analyses that included the largest number of studies were those of Zanatta et al. (72 studies) [38], Carra et al. (48 studies) [36] and Zhong et al. (36 studies) [30]. The most consulted databases were MEDLINE/PubMed, WOS, Scopus and the Cochrane Library. The most commonly used software for data analysis was RevMan 5.3. The most studied parameters were PPD, CAL, BOP and HbA1C. 

Two studies [27,32] evaluated serum TNF-α and IL-1β levels and two others [33,36] evaluated BMI. Only one of the studies [29] assessed the number of teeth lost during follow-up.

Most of the meta-analyses included studies in adult patients with periodontitis and T2DM.

The general and specific characteristics of the included meta-analyses are specified in Table 3 and Table 4.

### 3.2. Quality of Evidence (GRADE System)

The results of the quality of evidence for the included meta-analyses are shown in Table 5.

### 3.3. AMSTAR-2 Analysis

Almost all of the included studies showed considerable methodological errors. The studies by Yap et al. and Oliveira et al. [23,37] were the best rated and the study by Nicolini et al. [18] the worst. Domain 1 was fulfilled by most of the studies; however, the study by Nicolini et al. [22], despite following the PRISMA guidelines, did not cite the PICO format in the methodology section. Non-critical domains 5 and 6 (duplicate screening and the duplicity of data extractions) were the ones that created the greatest doubts in their interpretation. Regarding critical domains, domain 9 (the risk of bias of primary studies) was met by all studies; domains 11 (the appropriateness of methods used in meta-analysis) and 13 (the consideration of the risk of bias when interpreting results) were also met by most studies; however, those of Nicolini et al. [22], Elnour and Mirghani [33] and Zanatta et al. [38] were not clear in the reports. Critical domain 15 (the possible impact of publication bias) was respected by most studies, although some were assessed as unclear (Table 6). 

### 3.4. Analysis Using ROBIS Tool

**Figure 3:**
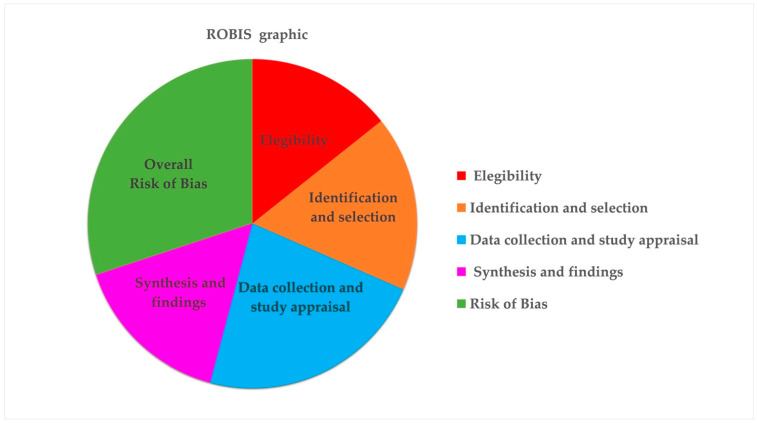
The ROBIS graph shows the most biased domains with different colors.

The results obtained from the evaluation using the ROBIS tool showed that the phase 3 domain (the risk of bias in review) was the most biased. The study by Elnour and Mirghani [33] obtained the worst evaluation, with unclear results in domains 1, 3 and 4 (study eligibility criteria, data collection and study appraisal and synthesis and findings, respectively) and high risk in domains 2 and phase 3 (the identification and selection of studies and the risk of bias in a review, respectively). The studies by Cao et al. [25] and Oliveira et al. [37] were the best rated, with a low risk of bias in all the domains considered by the assessment tool. The representative graph in Figure 3 shows the risk of bias in different colors (Figure 3 and Table 7). 

## 4. Discussion

It has been reported that approximately 22 new SRs appear every day [40] and that the synthesis of such a large amount of evidence requires the development of methodological tools capable of handling such a volume of documents and, at the same time, improving access to information with the aim of determining decision making by healthcare professionals [41].

In our study, we have used the term “review of systematic reviews” in the title to describe the evaluation of SRs with meta-analyses, in line with the most commonly used terminology and in accordance with the term used by Cochrane to describe a “review of systematic reviews” published in the Cochrane Library [42]. In short, SRs make it possible to summarize the results of several primary studies in a single article and to bring together the information available from several articles to strengthen the research; meta-analysis synthesizes, statistically, the data from a set of studies [43].

To our knowledge, this is the first time that a review of SRs with meta-analyses evaluating the effect of periodontal and adjuvant treatment, mainly on periodontitis and DM, has been performed, although other pathologies such as blood lipid profiles, nephropathy, non-alcoholic fatty liver disease and peri-implant pathologies were involved in the 18 meta-analyses included. We found that all included meta-analyses reported benefits of periodontal treatment on the periodontal parameters studied (PPD, CAL, BOP, GI, and PL) and some cytokines (IL-1β and TNF-α).

### 4.1. Antidiabetic Drugs

Regarding the use of adjuvants in combination with non-surgical periodontal therapy, discrepant results were reported. Nicolini et al. [22] reported benefits with the adjuvant use of antidiabetic drugs such as metformin. Regarding the efficacy of this hypoglycemic drug, Tao et al. [44] demonstrated, in vitro, that metformin inhibits the formation and activity of osteoclasts, so it could have a systemic beneficial effect on bone. Other studies have also shown that this drug would be able to palliate the alteration of the salivary microbiota caused by T2DM [45,46]. These contradictory results suggest that there are no clear conclusions demonstrating its clinical efficacy as an adjuvant therapy to non-surgical periodontal treatment.

### 4.2. Antimicrobial Drugs

Regarding the use of antimicrobials as adjuvant therapy, there were discrepancies in the meta-analyses: Yap et al. [23] evaluated the effect of systemic doxycycline in the treatment of diabetic patients with periodontitis and found no improvement in the levels of clinical adherence or reduction in HbA1C levels; on the contrary, Cao et al. [25] reported its effectiveness as an adjuvant to reduce HbA1c% in patients with periodontitis and T2DM. In this aspect, Das et al. [47], in a randomized clinical study on a sample of fifty-one diabetic subjects with chronic periodontitis, demonstrated that the addition of doxycycline to conventional periodontal therapy provides an additional benefit by reducing the glycemic level and improving periodontal health, and a recent study by Alblowi et al. [48] also found that treatment with azithromycin and doxycycline, as an adjunct to NSPT, could modulate host response and improve clinical outcomes in patients with T2DM and periodontitis; however, other studies also found no difference in glycemic control in T2DM patients with adjunctive systemic treatment with doxycycline [49]. Zanatta et al. [37] also studied the benefits of metronidazole as an adjuvant on periodontitis and glycemic control in patients with T2DM, although a recent SR found no difference with NSPT in combination with this antimicrobial [50]. The meta-analyses included in our review did not find unanimity on the benefit of the use of antimicrobials, as adjuvants to conventional periodontitis treatment, in producing a reduction in the glycemic level.

### 4.3. Lipid Metabolism

Garde et al. [24] found a weak association between the treatment of periodontitis in patients with T2DM and the reduction in triglyceride levels; in this regard, it has been shown in preclinical studies that periodontitis impairs lipid metabolism and excites atherosclerosis and that the treatment of periodontal disease improves the therapeutic efficacy of hyperlipidemia [51]; however, despite the possible correlation of periodontitis with serum lipid levels reported by several studies, a recent two-way Mendelian randomization analysis by Chen et al. [52] only identified an insignificant relationship between these levels and periodontitis; however, a recent study by Kudiyirickal and Pappachan [53] showed that elevated triglycerides were the second risk factor for periodontitis prevalence.

### 4.4. Biomodulation

Corbella et al. [27] analyzed the efficacy of systemic host modulatory factors, as adjuvants to NSPT, associated with reduced periodontal parameters and increased CAL, finding little evidence of a benefit. Despite these findings, a recent review by Deandra et al. [54] demonstrated that the use of certain probiotics helps to down-regulate the inflammatory process by up-regulating certain types of receptors and fatty acid production, directly targeting reactive oxygen species. They also indicated that melatonin could be an adjuvant of interest in the improvement in certain periodontal parameters such as PPD and GI, highlighting the need to reduce the prolonged systemic administration of antibiotics due to bacterial resistance problems.

The application of lasers to induce photobiomodulation effects has been studied as an adjuvant therapy to periodontal treatment for its ability to facilitate tissue healing, stimulate angiogenesis and reduce the inflammatory process [55]. Freire et al. [35] reported that a photobiomodulation adjunctive to periodontal treatment in individuals with T2DM contributes to the improvement in periodontal clinical parameters. However, controversy exists in this regard, and a narrative review by Theodoro et al. [56] noted that, although there are studies that have reported clinically beneficial effects of some lasers in periodontal treatment, there are few clinical reports on the additional benefits of lasers as adjunctive or complementary treatment. Despite these controversies, other meta-analyses [38,57] also found benefits on periodontal parameters in treatments using NSPT in combination with laser therapy. A modulation of the host response, in combination with the conventional treatment of periodontitis, appears to influence a curative response, and various therapies targeting cell signaling pathways, cytokines and enzymes are being developed to block the mechanisms responsible for periodontal tissue destruction.

Wu et al. [58], in a systematic review of 53 observational studies, definitively established a bidirectional association between T2DM and periodontitis, and in the same way, our study observed that periodontal treatment improves the clinical situation of both pathologies bidirectionally, which represents great advantages in clinical practice. This situation would be indicative for clinicians in establishing both background and adjunctive therapies to improve the health of patients with periodontitis and T2DM, as well as periodically assessing the glycemic control status of patients with T2DM and alerting them to the importance of comprehensive oral health assessment and care.

Both chronic pathologies present an inflammatory origin and share risk factors such as advanced age, low socioeconomic status, obesity, smoking, unhealthy diets and genetic predisposition to a deficient immune/inflammatory response [59]. Severe periodontal destruction has been explained by different mechanisms: subgingival plaque induces periodontal tissue degradation, and, in turn, subgingival plaque bacteria are associated with inflammation and insulin resistance; conversely, hyperglycemia can influence the subgingival microbiome, aggravating periodontitis [4,60]. Periodontal treatment reduces inflammatory cytokines and immune cells that aggravate T2DM, and hypoglycemic treatments minimize high-sugar environments that favor the development of certain dysbiosis-causing pathogens [61,62].

Our study has limitations that we would like to highlight: First, the eligibility criteria were limited to SRs with meta-analyses that evaluated the efficacy of periodontal treatment on periodontitis and DM, excluding others that evaluated its efficacy on other highly relevant systemic pathologies. Secondly, as in all secondary research, the quality of the results obtained will depend on the quality of the studies included and their risk of bias and methodological limitations. Therefore, we consider our conclusions to be limited.

As strengths, we could highlight that, to our knowledge, we have not found in the literature studies that evaluate, using the critical tools AMSTAR-2 and ROBIS, SRs with meta-analyses that determine the clinical evidence of periodontal treatment on periodontitis and diabetes, an aspect of great relevance in clinical practice. On the other hand, we also evaluated the methodological quality and risk of bias of the SRs using both tools.

## 5. Conclusions

Periodontal treatment and DM treatment contribute to improved clinical outcomes in a bidirectional manner.

However, despite the evidence for this claim, we have found a number of weaknesses in the included studies, using the AMSTAR-2 and ROBIS tools, mainly in terms of methodology and the risk of study bias. In addition, we have discussed the different opinions on adjuvant treatments to periodontal treatment with other studies, whether randomized clinical trials or SRs.

More critical studies highlighting the weaknesses of meta-analyses would be desirable and necessary to assist the clinician in decision making.

## Figures and Tables

**Table 1:** Search strategy for each consulted database.

Databases	Search Terms
PubMed/Medline	Periodontitis [Mesh] OR Periodontal AND Chronic Periodontitis [Mesh] OR Adult Periodontitis [Title/Abstract] OR Periodontal Disease* [Title/Abstract] OR Periodontal Pocket [Title/Abstract] OR Attachment Loss [Title/Abstract] OR Alveolar Bone Loss [Title/Abstract]; Periodontitis OR Etiology [Mesh]; Periodontitis AND Treatment [Mesh] OR Therapy OR Intervention [Mesh]; Periodontitis AND Diabetes Mellitus Type 2 OR T2DM [Text Word] OR Hyperglycemia [Text Word] OR Glycemic Control [Text Word] OR Glycosylated Hemoglobin [Text Word] OR Glycated Hemoglobin [Text Word].
Embase	Periodontitis OR Periodontal Disease* AND Therapy OR Treatment [Mesh] AND Diabetes OR Diabetes Mellitus/prevention & control* OR Metabolic Control OR Glycemic Control [Mesh] OR Glycated Hemoglobin OR HbA1c OR Systemic Inflammation AND Humans [Mesh].
Cochrane Central	Periodontitis OR Periodontal Disease* OR Periodontal Pocket OR Chronic Periodontitis OR Aggressive Periodontitis AND Periodontal Treatment [Mesh] OR Periodontal Therapy OR Periodontitis Therapy AND Diabetes mellitus Type 2 OR Hyperglycemia.
Dentistry & Oral Sciences	Periodontitis OR Periodontal Disease* AND Complications AND Treatment. Diabetes Mellitus Type 2 AND Periodontitis OR Periodontal Disease.
Web of Science	Periodontal Disease OR Periodontitis/Etiology AND Therapy AND Humans; Diabetes Complications* OR Hyperglycemia Complications; Diabetes Mellitus Type 2 Therapy OR Hyperglycemia Therapy.

**Table 2:** Inclusion and exclusion criteria.

Inclusion Criteria
1. SRs with meta-analyses performed in adult subjects (≥18 years) with a diagnosis of periodontitis and type 2 diabetes.
2. Outcome indicators: periodontal indices and/or glycemic level.
3. RCTs.
**Exclusion criteria**
1. SRs that did not include meta-analyses.
2. SR that used only one database in its bibliographic search strategy
3. Studies of predictive models or prognostic scales.

**Table 3:** General characteristics of the included meta-analyses.

Study, Year	RCTs Evaluated	Subjects Included	Type of Participants	Pathologies Involved	Diagnostic Criteria	Conclusions
Nicolini et al. 2019 [22]	3	206	Adult patients (aged 30+ years) diagnosed with chronic periodontitis	Periodontitis	NR	The adjuvant use of metformin provides additional benefit to the results of NSPT.
Yap and Pulikkotil 2019 [23]	6	276	Patients diagnosed with periodontitis and diabetes mellitus	Periodontitis and DM	NR	Systemic doxycycline as an adjunct to NSPT does not significantly improve clinical attachment levels or the reduction in HbA1C levels in the treatment of diabetic patients with periodontitis.
Garde et al. 2019 [24]	7	707	Individuals with T2DM	Periodontitis; T2DM; lipid profiles	NR	Reduction in triglyceride levels by approximately 8% achieved by periodontitis treatment in patients with T2DM.
Cao et al. 2019 [25]	14	629	Adult patients (aged ≥ 30 years) with periodontitis and T2DM	Periodontitis and T2DM	NR	Periodontal treatment with laser aPDT + doxycline is effective in reducing HbA1c% in periodontitis in non-smokers without severe T2DM complications.
Baeza et al. 2020 [26]	9	623	Patients ≥ 18 years with a diagnosis of T2DM and periodontitis	Periodontitis and T2DM	T2DM according to the WHO criteria; periodontitis as defined by the authors.	Evidence of periodontal treatment in improving metabolic control and reducing systemic inflammation in patients with T2DM.
Corbella et al. (**) 2021 [27]	27	2279	Patients ≥ 18 years affected by periodontitis, both systemically healthy and systemically compromised	Periodontitis and systemic diseases	Criteria extracted from included articles by 3 independent reviewers	There is no strong scientific evidence to advise the application in clinical practice of modulatory therapies such as laser or photomodulatory therapy as an adjunct to nonsurgical periodontal therapy.
Esteves de Lima et al. 2021 [28]	18	715	Individuals with T2DM	Periodontitis and T2DM	WHO criteria	NSPT modifies serum TNF-α levels at 6 months in patients with T2DM.
Zhao et al. 2021 [29]	9	376	Patients diagnosed with DM and Chronic Periodontitis	Periodontitis and DM	NR	DL + NSPT provided additional benefits on periodontal parameters (PD and CAL) and HbA1c levels compared to NSPT alone.
Zhong et al. 2022 [30]	36	3091	Patients with DM	DM; nephropathy; nonalcoholic fatty liver disease; periodontitis	NR	The results of this study indicate that antioxidant therapy is effective in the treatment of the above three complications of diabetes (periodontitis, nephropathy and nonalcoholic fatty liver disease).
Corbella et al. (*) 2023 [31]	11	504	Patients ≥ 18 years of age with previously untreated periodontitis affected by controlled or uncontrolled T2DM.	Periodontitis and T2DM	T2DM according to the World Health Organization criteria; periodontitis as defined by Papapanou et al. (2018) and Armitage (2004)	Decrease in short-term HbA1C (results should be interpreted with caution due to small effect sizes and statistical heterogeneity).
Da Silva-Junior et al. 2023 [32]	11	418	Individuals with T2DM and periodontitis	Periodontitis and T2DM	NR	aPDT as an adjunct to NSPT contributes significantly to the reduction in Periodontitis in individuals with type T2DM.
Elnour and Mirghani 2023 [33]	11	1469	NR	Periodontitis and T2DM	NR	There is a slight association between periodontal treatment and glycemic control among patients with uncontrolled diabetes. Early detection and treatment of periodontitis is recommended to improve glycemic control.
Greggianin et al. 2023 [34]	7	706	Adults with prediabetes or T2DM and periodontitis	Periodontitis and T2DM	NR	Absence of effect between NSPT and insulin resistance.
Freire et al. 2023 [35]	6	299	Individuals with T2DM and periodontitis	Periodontitis and T2DM	NR	Photobiomodulation complementary to contributes to the improvement in periodontal clinical parameters in individuals with T2DM, with further reduction in PPD and improvements in CAL.
Carra et al. 2023 [36]	48	909	Adult patients awaiting dental implant placement or having dental implants with peri-implant health	Peri-implant diseases	NR	Primary prevention of peri-implantitis is based on regular glycemic control. In diabetic patients receiving dental implants, glycemic control is essential for primary prevention of peri-implantitis.
Oliveira et al. 2023 [37]	11	1374	Patients with T2DM and periodontitis	T2DM; periodontitis	NR	Subgingival periodontal treatment produces a clinically relevant improvement in glycemic control in patients with T2DM and periodontitis.
Zanatta et al. 2024 [38]	72	1110	Adult subjects ≥ 18 years diagnosed with T2DM and Periodontitis	Periodontitis and T2DM	Definitions of T2DM and periodontitis described in the publication.	The adjuvant use of systemic metronidazole or ALA appears to bring significant additional benefits to both periodontitis and HbA1c reduction after NSPT in patients with T2DM.
Kang et al. 2024 [39]	10	566	Patients with periodontitis; patients with poorly controlled periodontitis and T2DM; and patients with controlled periodontitis and T2DM.	Periodontitis and T2DM	NR	NSPT had the best therapeutic effect in patients without T2DM.

(*), year 2023 publication; (**), year 2021 publication; T2DM, type 2 diabetes; WHO, World Health Organization; HbA1C, glycated hemoglobin; NR, does not report; aPDT, antimicrobial photodynamic therapy; DL, diode laser; ALA, alpha lipoic acid; SPT, surgical periodontal therapy; NSPT, non-surgical periodontal therapy; PPD, probing pocket depth.

**Table 4:** Specific characteristics of the included meta-analyses.

Study	Databases Consulted	Periodontal Intervention	Comparison	Intervention Effect	Meta-Analysis Software
Nicolini et al. [22]	MEDLINE-PubMed, Scopus and EMBASE	NSPT + metformin	NSPT alone or placebo	PPD; CAL; IBD	Stata 13.1
Yap and Pulikkotil [23]	PubMed and Scopus	NSPT + doxycycline	NSPT alone	PPD; CAL; HbA1C	RevMan 5.3
Garde et al. [24]	MEDLINE, EMBASE, PubMed, and WOS	Surgical or NSPT	NSPT or only supragingival scaling and polishing	PPD; BOP; TC; TG; LDL; HDL	NR
Cao et al. [25]	Pubmed, Embase, Cochrane Library and WOS	NSPT + adjuvant	No treatment	HbA1C	Network meta-analysis
Baeza et al. [26]	MEDLINE (PubMed) Cochrane Central Register of Controlled Trials	Conventional treatments, including oral hygiene instruction and SRP (with or without flap surgery)	Without periodontal treatment	HbA1C; CRP levels	RevMan 5.3
Corbella et al. (**) [27]	MEDLINE/Pubmed, Scopus, ISI Web of Science, EMBASE and Cochrane Central	NSPT protocol combined with a systemically delivered host-modulator drugs	NSPT alone or in association with placebo	PPD; CAL; GI; PL; TNF-α; IL-1β	RevMan 5.3
Esteves de Lima et al. [28]	PubMed, WOS, Scopus, Ovid and Lilacs	NSPT	Without periodontal treatment	BMI; HbA1C; Serum levels of TNF- α	RevMan 5.3
Zhao et al. [29]	MEDLINE, EMBASE, Cochrane Central Register WOS and Chinese BioMedical Literature	NSPT + DL	NSPT or placebo	PPD; CAL; BOP; PL; HbA1C	RevMan 5.3
Zhong et al. [30]	PubMed, Embase, The CENTRAL, WOS and Scopus	Antioxidant supplementation	NR	HbA1C; renal function; liver function; periodontitis	RevMan 5.4
Corbella et al. (*) [31]	MEDLINE/PubMed, Scopus, (OVID) EMBASE, and Cochrane Central	NSPT + LT + PDT	NSPT + Placebo	PPD; CAL; CEJ; HbA1C; BOP; Extracted teeth during follow-up	RevMan 5.3
Da Silva-Junior et al. [32]	PubMed, WOS, Cochrane, and LILACS	aPDT + NSPT	NSPT	BOP; PPD; CAL	NR
Elnour and Mirghani [33]	PubMed, Cochrane Library, and Google Scholar	NSPT	No treatment	HbA1C	RevMan 5.4
Greggianin et al. [34]	PubMed, Embase, WOS, Scopus, Cochrane and LILACS.	NSPT	Pre-intervention insulin resistance parameters or with a group that did not receive NSPT	CAL; PPD; BOP; BMI; HbA1C	RevMan 5.4
Freire et al. [35]	PubMed, WOS, Ovid, Cochrane, and LILACS	NSPT + Photobiomodulation	NSPT alone	BOP; PPD; CAL; HbA1C	NR
Carra et al. [36]	MEDLINE/PubMed, EMBASE, Cochrane Central Library, Base Search, Open Access Thesis and Dissertation (openthesis.org), and ClinicalTrials.gov	Preventive interventions	Patients who do not receive any preventive intervention	BOP; PPD; MBL	RevMan 5.3
Oliveira et al. [37]	MEDLINE/PubMed, Scopus, Embase, WOS, Latin American and Caribbean Health Science Information—LILACS, Livivo, DOSS (through EBSCO host), CINAHL (through EBSCO host) and Cochrane Library	SPT or NSPT	No subgingival intervention	HbA1C; BMI; PPD; CAL	NR
Zanatta et al. [38]	MEDLINE/ PubMed, EMBASE, Cochrane Central, WOS and LILACS	NSPT + Metronidazole or ALA	No periodontal treatment or NSPT alone	PPD; CAL; BOP; HbA1C	Network meta-analysis
Kang et al. [39]	PubMed, Embase, and the Cochrane Central Register of Controlled Trails	NSPT with OHI	Normal and systemically healthy control group (without diabetes) but diagnosed only with periodontitis	PPD; CAL; BOP	Stata 14.2

(*), year 2023 publication; (**), year 2021 publication; SRP, scaling and root planning; HbA1C, glycated hemoglobin; CRP, C-reactive protein; SPT, surgical periodontal treatment; NSPT, non-surgical periodontal treatment; OHI, oral hygiene instruction; LT, laser therapy; PDT, photodynamic therapy; PPD, probing pocket depth; CAL, clinical attachment level; BOP, bleeding on probing; GI, Gingival Index; PL, plaque level; TNF-α, Tumor Necrosis Factor-α; IL-1β, Interleukin-1β; BMI, Body Mass Index; DL, diode laser; ALA, alpha lipoic acid; NR, does not report; IBD, intrabony defect; TC, total cholesterol; TG, triglyceride; LDL, low-density lipoprotein; HDL, high-density lipoprotein; aPDT, antimicrobial photodynamic therapy; MBL, marginal bone level.

**Table 5:** GRADE assessment of meta-analysis.

Study	Quality Level
Nicolini et al. [22]	Moderate
Yap and Pulikkotil [23]	Very Low
Garde et al. [24]	Moderate
Cao et al. [25]	Moderate
Baeza et al. [26]	High
Corbella et al. (**) [27]	Low
Esteves de Lima et al. [28]	High
Zhao et al. [29]	Moderate
Zhong et al. [30]	High
Corbella et al. (*) [31]	Moderate
Da Silva-Junior et al. [32]	Moderate
Elnour and Mirghani [33]	High
Greggianin et al. [34]	Very Low
Freire et al. [35]	High
Carra et al. [36]	Moderate
Oliveira et al. [37]	Moderate
Zanatta et al. [38]	Moderate
Kang et al. [39]	Very Low

(*), year 2023 publication; (**), year 2021 publication.

**Table 6:** AMSTAR-2 assessment tool.

Study	Domain 1	Domain 2	Domain 3	Domain 4	Domain 5	Domain 6	Domain 7	Domain 8	Domain 9	Domain 10	Domain 11	Domain 12	Domain 13	Domain 14	Domain 15	Domain 16
Nicolini et al. [22]	O	O	O	O	O	O	O	O	O	O	O	O	O	O	O	O
Yap and Pulikkotil [23]	O	O	O	O	O	O	O	O	O	O	O	O	O	O	O	O
Garde et al. [24]	O	O	O	O	O	O	O	O	O	O	O	O	O	O	O	O
Cao et al. [25]	O	O	O	O	O	O	O	O	O	O	O	O	O	O	O	O
Baeza et al. [26]	O	O	O	O	O	O	O	O	O	O	O	O	O	O	O	O
Corbella et al. [27]	O	O	O	O	O	O	O	O	O	O	O	O	O	O	O	O
Esteves Lima et al. [28]	O	O	O	O	O	O	O	O	O	O	O	O	O	O	O	O
Zhao et al. [29]	O	O	O	O	O	O	O	O	O	O	O	O	O	O	O	O
Zhong et al. [30]	O	O	O	O	O	O	O	O	O	O	O	O	O	O	O	O
Corbella et al. [31]	O	O	O	O	O	O	O	O	O	O	O	O	O	O	O	O
Da Silva-Junior et al. [32]	O	O	O	O	O	O	O	O	O	O	O	O	O	O	O	O
Elnour and Mirghani [33]	O	O	O	O	O	O	O	O	O	O	O	O	O	O	O	O
Greggianin et al. [34]	O	O	O	O	O	O	O	O	O	O	O	O	O	O	O	O
Freire et al. [35]	O	O	O	O	O	O	O	O	O	O	O	O	O	O	O	O
Carra et al. [36]	O	O	O	O	O	O	O	O	O	O	O	O	O	O	O	O
Oliveira et al. [37]	O	O	O	O	O	O	O	O	O	O	O	O	O	O	O	O
Zanatta et al. [38]	O	O	O	O	O	O	O	O	O	O	O	O	O	O	O	O
Kang et al. [39]	O	O	O	O	O	O	O	O	O	O	O	O	O	O	O	O

O Yes; O no; O partial yes.

**Table 7:** Assessment of concerns with the review process and risk of bias in the eighteen SRs included in the review. ROBIS results.

	Phase 2 Concerns with Review Process				Phase 3 Risk of Bias in Review
Study	Domain 1 (Study Eligibility Criteria)	Domain 2 (Identification and Selection of Studies)	Domain 3 (Data Collection and Study Appraisal)	Domain 4 (Synthesis and Findings)	
Nicolini et al. [22]	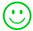	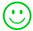	**?**	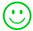	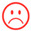
Yap and Pulikkotil [23]	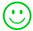	**?**	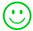	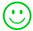	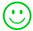
Garde et al. [24]	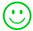	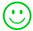	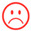	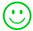	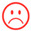
Cao et al. [25]	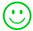	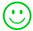	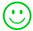	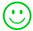	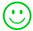
Baeza et al. [26]	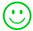	**?**	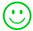	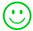	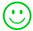
Corbella et al. [27]	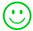	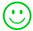	**?**	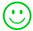	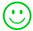
Esteves Lima et al. [28]	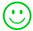	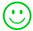	**?**	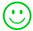	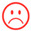
Zhao et al. [29]	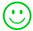	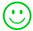	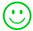	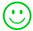	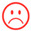
Zhong et al. [30]	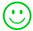	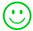	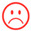	**?**	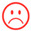
Corbella et al. [31]	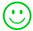	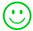	**?**	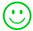	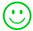
Da Silva-Junior et al. [32]	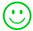	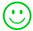	**?**	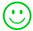	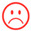
Elnour and Mirghani [33]	**?**	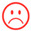	**?**	**?**	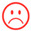
Greggianin et al. [34]	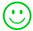	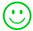	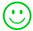	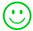	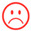
Freire et al. [35]	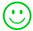	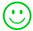	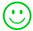	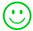	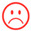
Carra et al. [36]	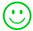	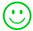	**?**	**?**	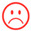
Oliveira et al. [37]	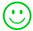	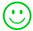	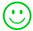	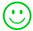	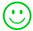
Zanatta et al. [38]	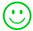	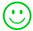	**?**	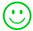	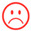
Kang et al. [39]	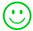	**?**	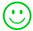	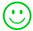	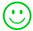

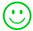
 Low risk; 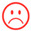 High risk; **?** Unclear risk.

## Supplementary Materials

The following supporting information can be downloaded at: https://www.mdpi.com/article/10.3390/healthcare12181844/s1, Table S1: PRISMA check list.
