# Supplementary material for: Effect of Periodontal Treatment in Patients with Periodontitis and Diabetes: Review of Systematic Reviews with Meta-Analyses in the Last Five Years
Source: Healthcare (Basel). 2024 Sep 14;12(18):1844. doi: 10.3390/healthcare12181844 (PMC11431200; doi:10.3390/healthcare12181844)
Supplement: Supplementary file 1 [file healthcare-12-01844-s001.zip › healthcare-3127360-supplementary.pdf]

Supplementary Material

Table S1. PRISMA Check list.

Section and Topic	Item #	Checklist item	Location where item is reported
**TITLE**			
Title	1	Effect of periodontal treatment in patients with periodontitis and diabetes: Review of Systematic Reviews with Meta-analyses in the last five years.	1
**ABSTRACT**			
Abstract	2	Periodontitis is a chronic infectious-inflammatory pathology, with a high prevalence, which destroys the dental support and, if left untreated, leads to tooth loss. It is associated with other pathologies, particularly diabetes mellitus. Our objective was to conduct a review of systematic reviews with meta-analyses to determine the evidence for periodontal treatment on periodontitis and diabetes. Second, to assess the risk of bias and methodological quality using the AMSTAR-2 and ROBIS tools. We performed bibliographic searches in PubMed/Medline, Embase, Cochrane Central, Dentistry & Oral Sciences Source databases and in the Web of Science (WOS) scientific information service to identify systematic reviews with meta-analyses from the last five years. Eighteen studies that met the inclusion criteria and evaluated 16,247 subjects were included. The most studied parameters were Probing Pocket Depth, Clinical Attachment Level, Bleeding on Probing and the Glycated Hemoglobin. Most of the included meta-analyses evaluated adult patients with periodontitis and Type 2 Diabetes Mellitus (T2DM). Most of the meta-analyses considered and assessed by AMSTAR-2 showed significant methodological errors. Risk of bias was the domain with the worst assessment with the ROBIS tool. Despite the weaknesses of the included meta-analyses in terms of methodological quality and risk of bias, periodontal treatment and DM treatment appear to contribute to improved clinical outcomes in a bidirectional manner between periodontitis and DM.	1
**INTRODUCTION**			
Rationale	3	In the last decades numerous investigations have studied the association of periodontitis with other systemic pathologies, such as diabetes, cardiovascular diseases, metabolic bone pathologies, premature birth and, recently, Alzheimer's disease, as well as with other inflammatory and oncological pathologies.<br>Diabetes is the pathology most closely related to periodontitis and it is well known that patients with this pathology, especially in uncontrolled situations, are at high risk of developing periodontitis, as well as the impact of periodontitis on the glycemic control of diabetes mellitus, although a recent Cochrane review showed only moderate certainty that periodontal treatment improves glycemic control compared to untreated individuals.	2
Objectives	4	The aim of our work was to perform a review of SRs with meta-analyses, including randomized clinical studies, to determine the evidence of periodontal treatment on periodontitis and diabetes. Our secondary objective was to assess the methodological quality and risk of bias of the SRs using the AMSTAR-2 and ROBIS tools.	3
**METHODS**			
Eligibility criteria	5	Table 2	5
Information sources	6	Table 1	4
Search strategy	7	Table 1	4
Selection process	8	Table 2	5
Data collection process	9	The We conducted an electronic search in PubMed/Medline, Embase, Cochrane Central, Dentistry & Oral Sciences Source databases and in the Web of Science (WOS) scientific information service to identify SRs with meta-analyses published in English during the last five years, using the EndNote bibliographic reference manager (Clarivate Analytics).<br>The search strategy was designed with the help of an expert documentalist using the terms described in Table 1. We also searched the gray literature to obtain as much information as possible and to avoid bibliographic bias (GreyNet International).<br>Two independent reviewers (NL-V and JAB-R) collected the titles and abstracts of the selected articles and entered them into an Excel spreadsheet, and subsequently assessed the methodological quality of the included meta-analyses using the AMSTAR-2 tool and the risk of bias using the ROBIS tool. Discrepancies were resolved by consensus or by the intervention of an external evaluator. A narrative description of the extracted data was prepared, analysed and an overlap of evidence document was prepared by cross-checking. A minimum overlap was considered to be between 0-5%, moderate between 6-10%, high 11-15% and very high when it exceeded 15%.	4
Data items	10a	List and define all outcomes for which data were sought. Specify whether all results that were compatible with each outcome domain in each study were sought (e.g. for all measures, time points, analyses), and if not, the methods used to decide which results to collect.	----
	10b	List and define all other variables for which data were sought (e.g. participant and intervention characteristics, funding sources). Describe any assumptions made about any missing or unclear information.	----
Study risk of bias assessment	11	GRADE criteria	4
Effect measures	12	Specify for each outcome the effect measure(s) (e.g. risk ratio, mean difference) used in the synthesis or presentation of results.	----
Synthesis methods	13a	Describe the processes used to decide which studies were eligible for each synthesis (e.g. tabulating the study intervention characteristics and comparing against the planned groups for each synthesis (item #5)).	----

Section and Topic	Item #	Checklist item	Location where item is reported
	13b	Describe any methods required to prepare the data for presentation or synthesis, such as handling of missing summary statistics, or data conversions.	----
	13c	Describe any methods used to tabulate or visually display results of individual studies and syntheses.	----
	13d	Describe any methods used to synthesize results and provide a rationale for the choice(s). If meta-analysis was performed, describe the model(s), method(s) to identify the presence and extent of statistical heterogeneity, and software package(s) used.	----
	13e	Describe any methods used to explore possible causes of heterogeneity among study results (e.g. subgroup analysis, meta-regression).	----
	13f	Describe any sensitivity analyses conducted to assess robustness of the synthesized results.	----
Reporting bias assessment	14	Describe any methods used to assess risk of bias due to missing results in a synthesis (arising from reporting biases).	----
Certainty assessment	15	Describe any methods used to assess certainty (or confidence) in the body of evidence for an outcome.	----
**RESULTS**			
Study selection	16a	The main search identified 765 records in the last 5 years up to March 2024. The results were imported into Mendeley to remove duplicate and non-useful records for our study, resulting in 66 records for analysis. Subsequently, 43 were removed due to full-text inaccessibility and different results being reported, leaving 23 full-text records for as-sessment. Finally, for different reasons, 5 more were removed, resulting in 18 studies for inclusion.	5,6
	16b	The results were imported into Mendeley to remove duplicate and non-useful records for our study, resulting in 66 records for analysis. Subsequently, 43 were removed due to full-text inaccessibility and different results being reported, leaving 23 full-text records for assessment. Finally, for different reasons, 5 more were removed	5
Study characteristics	17	Tables 3 and 4	7-12
Risk of bias in studies	18	Table 5	12
Results of individual studies	19	For all outcomes, present, for each study: (a) summary statistics for each group (where appropriate) and (b) an effect estimate and its precision (e.g. confidence/credible interval), ideally using structured tables or plots.	----
Results of syntheses	20a	For each synthesis, briefly summarise the characteristics and risk of bias among contributing studies.	----
	20b	Present results of all statistical syntheses conducted. If meta-analysis was done, present for each the summary estimate and its precision (e.g. confidence/credible interval) and measures of statistical heterogeneity. If comparing groups, describe the direction of the effect.	----
	20c	Present results of all investigations of possible causes of heterogeneity among study results.	----
	20d	Present results of all sensitivity analyses conducted to assess the robustness of the synthesized results.	----
Reporting biases	21	Present assessments of risk of bias due to missing results (arising from reporting biases) for each synthesis assessed.	----
Certainty of evidence	22	Present assessments of certainty (or confidence) in the body of evidence for each outcome assessed.	----
**DISCUSSION**			
Discussion	23a	We found that all included meta-analyses re-ported benefits of periodontal treatment on the periodontal parameters studied (PPD, CAL, BOP, GI, PL) and some cytokines (IL-1β and TNF-α).	17
	23b	Our study has limitations that we would like to highlight: First, the eligibility criteria were limited to SR with meta-analyses that evaluated the efficacy of periodontal treatment on periodontitis and DM, excluding others that evaluated its efficacy on other highly relevant systemic pathologies. Secondly, as in all secondary research, the quality of the results obtained will depend on the quality of the studies included and their risk of bias and methodological limitations. Therefore, we consider our conclusions to be limited.	19
	23c	Discuss any limitations of the review processes used.	----
	23d	Discuss implications of the results for practice, policy, and future research.	----
**OTHER INFORMATION**			
Registration and protocol	24a	The previously elaborated protocol was registered in INPLASY, registration number: INPLASY202450078; DOI number: 10.37766/inplasy2024.5.0078.	3
	24b	Indicate where the review protocol can be accessed, or state that a protocol was not prepared.	----
	24c	Describe and explain any amendments to information provided at registration or in the protocol.	----
Support	25	Non-financial support for the review	20
Competing interests	26	The authors declare no conflicts of interest	20
Availability of data, code and other materials	27	Report which of the following are publicly available and where they can be found: template data collection forms; data extracted from included studies; data used for all analyses; analytic code; any other materials used in the review.	----
